# Availability of First-line Therapies for Termination of Status Epilepticus on the General Adult, Old Age and Forensic Wards at Woodland View Hospital

**DOI:** 10.1192/bjo.2025.10575

**Published:** 2025-06-20

**Authors:** Anna Dobrowolska

**Affiliations:** Woodland View Hospital, NHS Ayrshire and Arran, Irvine, United Kingdom

## Abstract

**Aims:** To ensure adequate availability and stocking of benzodiazepines on the wards for management of status epilepticus.

To ensure ward staff can locate treatment for status epilepticus during a medical emergency.

To ensure current ward staff are up to date with local ALS guidelines on status epilepticus.

**Methods:** Three general adult wards, two old age wards, and one intensive psychiatric care unit were audited. Two cardiac arrest packs were also audited as these are part of the cardiac arrest team protocol in Woodland View Hospital. Ward and pack audits occurred at random, and wards were not informed prior. Each ward was checked for medication availability, dosage available and expiration date.

Benzodiazepines were checked for as per the ALS guidelines; this included lorazepam, diazepam, and midazolam. These preparations were intravenous lorazepam 2–4 mg, rectal diazepam 10 mg, and buccal midazolam 10 mg.

Following the first cycle of data collection, a questionnaire was distributed to the ward team to identify gaps in ward knowledge of benzodiazepine location on the ward and status epilepticus management.

The intervention was a teaching session which was held with the ward staff to help improve staff confidence with identifying status epilepticus, medications, as well as timely management of seizures on the wards.

A second cycle of data collection and questionnaires was completed.

**Results:** The results showed that intravenous medications (lorazepam) are not regularly supplied at Woodland View. This was due to lack of IV training amongst the nursing staff.

All wards (100%) were adequately stocked with a supply of rectal diazepam which was the correct dose and in date. Buccal midazolam was only available in two (33%) of the six wards audited. The emergency packs had two preparation options of either midazolam or diazepam which were both in date with the correct dosing.

Following the intervention of staff education, ward staff self-reported that they felt more comfortable with identifying a seizure and locating medications in a timely fashion. There was a 50% self-reported improvement in staff’s ability to identify the medications required for seizure management and a 19.5% self-reported improvement in staff’s confidence of locating medications.

**Conclusion:** This audit demonstrated that first-line benzodiazepines for the treatment of status epilepticus are available on all wards in at least one preparation. It highlighted the need for staff education about these preparations and their location on the wards. Staff self-reported an improvement in their confidence and knowledge of status epilepticus following the intervention.

